# Harmony of Protein Tags and Chimeric Molecules Empowers Targeted Protein Ubiquitination and Beyond

**DOI:** 10.3390/cells13050426

**Published:** 2024-02-28

**Authors:** Aggie Lawer, Luke Schulz, Renata Sawyer, Xuyu Liu

**Affiliations:** 1 School of Chemistry, Faculty of Science, The University of Sydney, Camperdown, NSW 2050, Australia; 2 Heart Research Institute, The University of Sydney, Newtown, NSW 2042, Australia

**Keywords:** post-translational modification, ubiquitination, PROTAC, dTAG, proximity, phosphorylation, acetylation

## Abstract

Post-translational modifications (PTMs) are crucial mechanisms that underlie the intricacies of biological systems and disease mechanisms. This review focuses on the latest advancements in the design of heterobifunctional small molecules that hijack PTM machineries for target-specific modifications in living systems. A key innovation in this field is the development of proteolysis-targeting chimeras (PROTACs), which promote the ubiquitination of target proteins for proteasomal degradation. The past decade has seen several adaptations of the PROTAC concept to facilitate targeted (de)phosphorylation and acetylation. Protein fusion tags have been particularly vital in these proof-of-concept studies, aiding in the investigation of the functional roles of post-translationally modified proteins linked to diseases. This overview delves into protein-tagging strategies that enable the targeted modulation of ubiquitination, phosphorylation, and acetylation, emphasizing the synergies and challenges of integrating heterobifunctional molecules with protein tags in PTM research. Despite significant progress, many PTMs remain to be explored, and protein tag-assisted PTM-inducing chimeras will continue to play an important role in understanding the fundamental roles of protein PTMs and in exploring the therapeutic potential of manipulating protein modifications, particularly for targets not yet addressed by existing drugs.

## 1. Introduction

### 1.1. Protein Post-Translational Modifications (PTMs)

Post-translational modifications, which entail either the covalent addition of new functional groups or the removal of existing ones from amino acid residues in proteins, play a pivotal role as regulators in almost every physiological process. They profoundly influence protein structure, function, and stability, serving as nature’s tools to amplify biological complexity far beyond the scope of the cellular genome [1,2,3,4]. To date, over 620 types of PTMs [5] and 87,308 experimentally identified PTMs [6] have been discovered [7], the most common of which include phosphorylation [8], ubiquitination [9,10], acetylation [11,12], and sulfation [13,14]. The library of modified proteins is further expanded by the ability to include multiple PTMs on a single protein, where each unique combination can offer granular control over the protein’s properties. Dysregulated PTM processes have been implicated in a wide range of pathological conditions, spanning from carcinogenesis and cardiovascular disease to infectious diseases caused by viruses and bacteria [5,15,16,17,18,19,20,21,22]. As such, targeting PTM events has become an increasingly attractive approach in the development of contemporary targeted therapies. Although the significance of certain protein PTMs has been documented in isolated studies [23,24], our understanding of the functional roles of PTMs across the wider proteome is still in its infancy. Several challenges continue to hamper both the fundamental research into and therapeutic application of protein PTMs. The primary obstacle lies in determining the direct, unequivocal link between a site-specific PTM and its effect on protein function and ensuing cellular phenotype [25,26,27]. While genetic techniques such as site-directed mutagenesis offer some insights, they fall short of fully capturing the dynamic and complex nature of PTMs. This shortfall arises as the techniques introduce an amino acid mutation that cannot be modified by cellular enzymes, making it difficult to study rapid, reversible PTM events and gain-of-function phenotypes that PTMs might prompt in real time [28,29]. Moreover, given that many proteins undergo various PTMs at different sites, achieving a homogeneous form of PTM becomes essential for unambiguous functional interpretation [30,31]. Such homogeneity can be achieved through in vitro chemical or semi-synthetic synthesis strategies but is a formidable challenge for in vivo studies [32,33,34].

Considerable efforts have also been devoted to developing inhibitors or activators capable of modulating the activity of specific PTM machinery to achieve targeted PTMs in living systems. Eighty-eight small-molecule drugs, including kinase, deacetylase, and methyltransferase inhibitors, have received regulatory approval for cancer treatment [35], with many more in the pipeline. However, challenges still exist in targeted PTM research, as exemplified by the development of kinase inhibitors [36,37]. The kinases targeted in cancer treatments often have a broad substrate range beyond the intended specific substrate and signaling pathway. As a result, chemically inhibiting kinase activity impacts a wide array of protein phosphorylation events, limiting their effectiveness in precisely interrogating a specific event of interest [37].

### 1.2. Chemically Induced Proximity Enabling Targeted Protein (De)ubiquitination

Chemically induced proximity (CIP) has become a significant method for investigating protein–protein interactions (PPIs). CIP involves using small molecules to draw two proteins closer together, thereby facilitating their intermolecular interactions and chemical reactions. A prime example of this approach is proteolysis-targeting chimeras (PROTACs), which demonstrates the vast potential of CIP and related therapeutic strategies in the study of targeted ubiquitination PTMs.

PROTACs are heterobifunctional molecules equipped with dual functional ligands that selectively bind to a protein of interest (POI) and an E3 ubiquitin ligase. This unique configuration facilitates the formation of a ternary complex comprising the PROTAC molecule and both proteins. The resulting proximity-driven interactions between the two macromolecules catalyze the ubiquitination of the POI, which involves attaching an 8.6 kDa ubiquitin protein; the E3 ligase continues the catalysis of ubiquitin transfer from an E2 ligase to the prior ubiquitin molecule leading to the formation of a polyubiquitin chain [38]. Such polyubiquitin chains typically form through isopeptide bonds connecting the C-terminal glycine (Gly) of one ubiquitin to lysine residue 48 (Lys48) of another ubiquitin within the chain. This process, known as Lys48-linked polyubiquitination, marks a protein for degradation by the proteasome, the cellular apparatus tasked with breaking down proteins tagged with a polyubiquitin chain [39]. This system is primarily effective in degrading cytosolic proteins and less efficient for transmembrane or extracellular proteins [40]. Instead, for these membrane proteins, ubiquitination typically results in altered localization—mostly via lysine residue 63 (Lys63)-linked polyubiquitination—driving their internalization into endosomes [40,41,42]. PROTAC technology opens new avenues to address “undruggable” protein targets since binding to any site on the POI (not necessarily the active site), in principle, suffices for the formation of the PROTAC-mediated ternary complex. Furthermore, the approach provides a new dimension of target selectivity, derived from the unique manner in which the ternary complex is assembled. The spatial configuration of this complex will be more cooperative for certain pairs of POI and E3 ligase, translating into more efficient ubiquitin transfer and rapid degradation of the POI, even compared to the POI’s homologues in the same family [43]. The emerging role of PROTAC modality in therapeutic innovation is highlighted by the 26 clinical trials utilizing targeted protein degradation (TPD) approaches for cancers [44,45,46]. Several reviews have offered comprehensive discussions on PROTAC discovery, synthesis, and application in therapeutic research [44,47,48,49,50,51,52,53].

**Figure 1:**
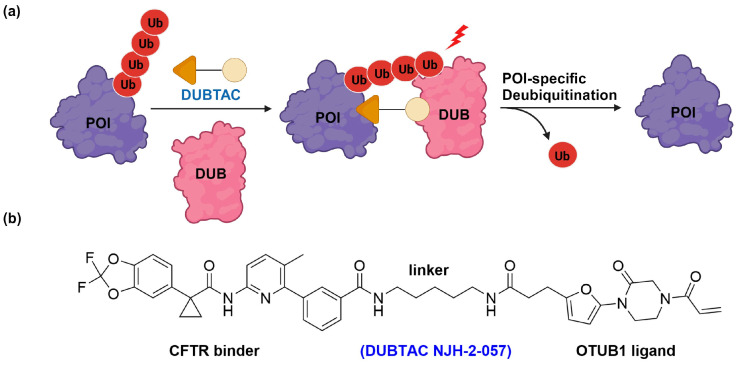
Proximity-induced deubiquitination mediated by DUBTAC. (**a**) Mechanism of DUBTAC in deubiquitination and stabilization of the target protein; (**b**) structure of DUBTAC NJH-2-057 consisting of CFTR and OTUB1 ligands.

Henning et al. recently introduced a novel category of chimeric molecules termed deubiquitinase-targeting chimeras (DUBTACs), designed to enhance the stability of protein targets by aiding in the removal of ubiquitin PTMs (Figure 1) [54]. Similar in concept to PROTACs, DUBTACs are heterobifunctional molecules that bring a deubiquitinase enzyme, such as OTU Deubiquitinase, Ubiquitin Aldehyde Binding 1 (OTUB1), into proximity with the target protein. This initiates the enzymatic hydrolysis of the isopeptide bonds between protein Lys and ubiquitin proteins, thereby reducing the rate of proteasomal degradation and increasing the lifetime of POI within the cell. Although there are only a few examples so far, DUBTACs have demonstrated their efficacy in stabilizing the protein level of cystic fibrosis transmembrane conductance regulator (∆F508-CFTR), leading to enhanced chloride channel conductance in human cystic fibrosis bronchial epithelium [54]. DUBTACs were also successfully tested against a tumor suppressor kinase essential in non-malignant eukaryotic somatic cells known as WEE1. In a recent advancement, Liu et al. have developed an innovative variant of DUBTAC, named transcription factor DUBTAC (TF-DUBTAC) [55]. This new type integrates a DNA oligonucleotide, which targets a specific transcription factor (TF), with a covalent small-molecule ligand for OTUB1. The aim is to enhance the stability of tumor suppressor transcription factors, protecting them from ubiquitin-mediated degradation.

While the full therapeutic potential of these DUBTAC methodologies is still under exploration, these initial studies pave the way for further investigation into the functional roles of proteins regulated by the ubiquitin–proteasome system (UPS), offering exciting prospects for future research and applications.

Although PROTACs and DUBTACs are proven to efficiently modify POI levels inside the cell, there are many other forms of CIP which are designed for modifying extracellular and transmembrane protein levels, such as lysosome-targeting chimeras (LYTACs) [56], proteolysis targeting antibodies (PROTABs) [57], and cytokine-receptor-targeting chimeras (KineTACs) [58], while autophagosome-tethering compounds (ATTECs) [59,60,61] and autophagy-targeting chimeras (AUTACs) [62] concentrate on autophagosome-mediated protein degradation. LYTACs induce proximity between an extracellular POI and cell surface receptors involved in protein internalization, which allows these POIs to be internalized and exposed to lysosomal degradation. PROTAB, a bispecific antibody, leverages Zinc and Ring Finger 3, a surface E3 ubiquitin ligase, to degrade an array of transmembrane proteins in a manner reminiscent of traditional PROTACs. KineTACs function as another form of bispecific antibodies, having one arm designed to bind specifically to a cytokine receptor, while the other arm is engineered to attach to the POI. By harnessing the natural internalization mechanisms inherent in cytokine receptors, KineTACs facilitate the linkage of target proteins to this receptor-mediated internalization process. This strategic connection also directs the target proteins towards lysosomal degradation. Recently, some protein degraders [63], have been engineered to directly usher protein targets into the proteasome without the need of recruiting a distinct E3 ligase.

Yet, all of the bifunctional molecules above are relatively one-dimensional, acting as TPD or protein stabilizer. This makes them less useful when targeting POIs that manifest multiple states of function for different biological processes, including when modifications other than ubiquitination are required to effect the desired phenotype. Moreover, conventional PROTAC and similar targeting chimera technologies are constrained by the limited availability of small-molecule ligands for POIs, as only 20% of the human proteome has been liganded [64]. To overcome this challenge, researchers have developed innovative approaches that utilize protein tags in conjunction with small-molecule or peptide probes. These methods focus on catalyzing targeted PTMs by strategically localizing the heterobifunctional molecule or molecular glue onto a protein tag (fused to the POI genetically), which subsequently orchestrates the recruitment of the necessary PTM enzymatic machinery and execution of PTMs on the POI. One of the early approaches utilized the plant E3 ligase F-box transport inhibitor response 1 (TIR1), exogenously expressed in non-plant cells. This approach triggered the ubiquitination and subsequent degradation of proteins tagged with an Auxin-inducible degron (AID) upon the addition of the plant hormone Auxin [65]. Subsequent advancements have seen the degradation of HaloTag fusion proteins using hydrophobic tagging [66] or HaloTag-targeted PROTACs (HaloPROTACs) [67,68,69]. Another notable approach involves the degradation tag (dTAG) system, a phthalimide-based chimeric compound that targets proteins fused with a FK506-binding protein (FKBP) mutant tag, inducing degradation via the protein cereblon (CRBN) or Von Hippel–Lindau disease tumor suppressor (VHL) E3 ligase [70,71].

**Figure 2:**
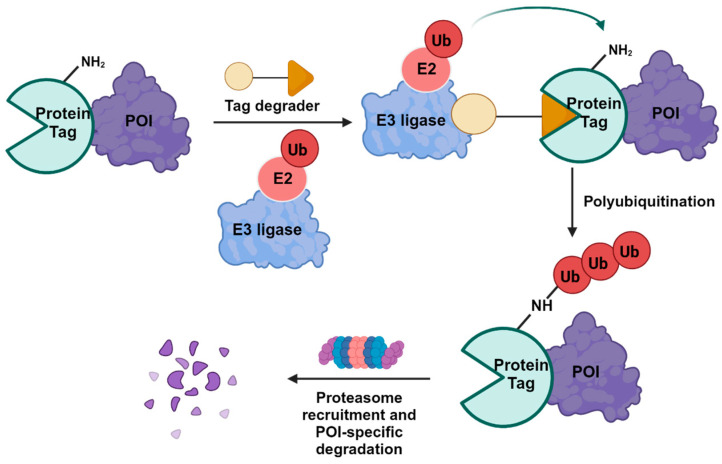
Mechanisms of action of tagged protein degraders.

This review explores the integration of protein tag fusion systems with the principle of CIP (Figure 2), showcasing their efficacy in facilitating targeted PTMs. While the primary focus is on ubiquitination modifications, the scope of this discussion extends to other PTMs, such as phosphorylation and acetylation. Furthermore, this review also provides a brief analysis of the inherent advantages and limitations associated with these protein/peptide-tagging systems.

## 2. HaloTag-Assisted Targeted Protein Modifications

### 2.1. HaloTag Technology

**Figure 3:**
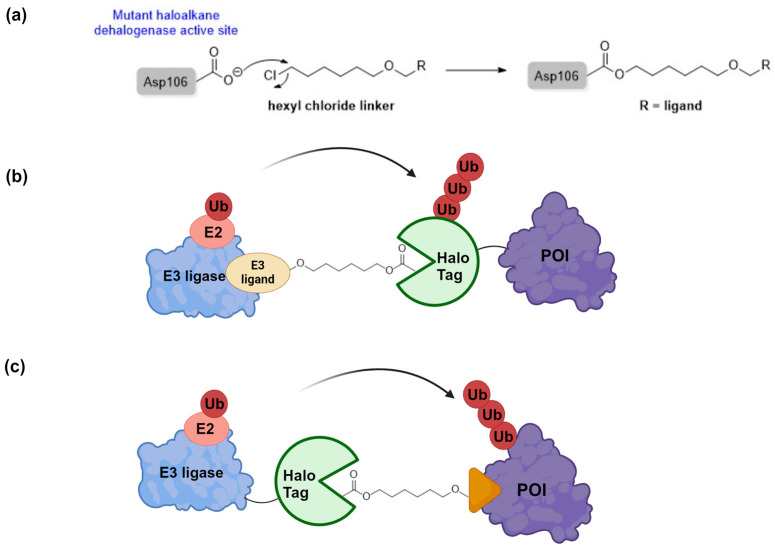
The application of HaloTag technology in targeted protein degradation. (**a**) Nucleophilic displacement of terminal chloride with Asp106 resulting in a highly stable covalent adduct. The two different modalities of HaloPROTAC constructs using (**b**) Halo POI fusion or (**c**) Halo E3 ligase fusion, respectively.

HaloTag, derived from bacterial dehalogenase, is a widely used protein tag engineered to covalently bind synthetic ligands containing a hexyl chloride motif (Figure 3a) [72,73]. This innovative platform allows for the fusion of HaloTag with target proteins, enabling the comprehensive analysis of protein function and interactions using a single genetic construct. To enhance stability, the haloalkane dehalogenase component has been modified to react with halogenated substrates, such as hexyl chloride, irreversibly. During enzyme–ligand interaction, a nucleophilic displacement occurs, forming an ester bond by replacing the terminal chloride with the carboxylate side chain of aspartate residue 106 (Asp106). Mutating histidine residue 272 (His272) in wild-type haloalkane dehalogenase to a phenylalanine residue (Phe272) prevents hydrolysis, leading to a stable covalent adduct that remains orthogonal to the human proteome (Figure 3a). Such covalent interaction is completely orthogonal to the human proteome. A comprehensive library of human proteins fused with HaloTag is commercially available, facilitating functional studies utilizing this tagging system [74,75].

There are many advantages of utilizing HaloTag over traditional protein-tagging systems [72]. The binding between the HaloTag protein and its hexyl chloride ligand is rapid, irreversible, and highly specific, resulting in near-complete occupancy of the Halo protein in less than 10 min [76,77]. The estimated rate constant for the reaction between HaloTag and its chloroalkane substrate is 2.7 × 10^6^ M^−1^ s^−1^ [76]. Finally, the cell penetration efficiency of HaloTag-targeted molecules can be easily assessed through competitive assay against a chloroalkane-modified fluorophore [78].

There have been two major revisions of HaloTag fusion proteins. HaloTag2 was an early version of the HaloTag system, and hydrophobic tags have been developed to degrade these HaloTag2 fusion proteins [66]. In contrast, the current commercially available version is the HaloTag7 fusion protein. HaloTag7 differs from HaloTag2 by several amino acids and offers increased protein stability, as it is not susceptible to degradation by these hydrophobic tags [74,75]. Unless specified otherwise, any references to “HaloTag” in the subsequent discussion will pertain to the latest HaloTag7 technology.

The emergence of HaloTag has had a significant impact on synthetic biology and protein engineering, leading to diverse applications in chemical biology research, such as protein isolation and purification [79], in vitro and in vivo imaging [76,80], and functional interactome studies [81]. One of the most prevalent applications lies in its use as a method for attaching fluorescent markers, which facilitates the real-time tracking of target proteins within living cells. Such biomedical applications of HaloTag technology have been extensively discussed elsewhere [72,82].

### 2.2. HaloPROTAC

To fully harness the potential of TPD in biological research, HaloTag fusion POIs have been introduced to eliminate the necessity for a specific binder or inhibitor in the creation of PROTAC molecules [28,83,84,85,86,87,88]. HaloPROTACs are pioneers in this category and possess a distinct hexyl chloride motif and an E3 ligase ligand (Figure 3b,c). Upon forming a covalent bond with the Halo protein, HaloPROTACs attract an E3 ligase, which aids in the ubiquitination and subsequent degradation of the HaloTag-fused POI. In theory, the use of the commercially available HaloTag fusion protein library enables the investigation of HaloPROTAC-mediated degradation of any human protein, despite a requisite 1:1 HaloTag/HaloPROTAC ratio.

**Figure 4:**
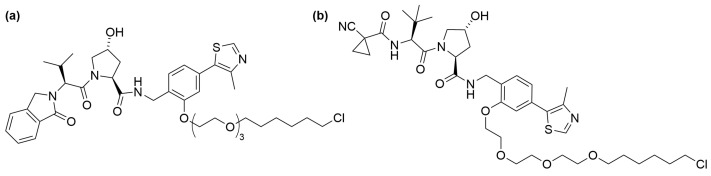
Structures of (**a**) HaloPROTAC 3 and (**b**) HaloPROTAC E.

HaloPROTAC 3 is distinguished as one of the most effective members of the HaloPROTAC series. This molecule features a VHL E3 ligase ligand linked to a hexyl chloride via a PEG3 linker, as depicted in Figure 4a. Its potency is evidenced by a DC_50_ (half-maximal degradation concentration) of 19 nM against a HaloTag fusion of enhanced green fluorescent protein (EGFP) [67]. Additionally, it also demonstrated degradation of cytoplasmic proteins such as extracellular signal-regulated protein kinase 1 (ERK1) or MAP/ERK kinase 1 (MEK1) at similar nanomolar concentrations. Comparative studies between HaloPROTAC3 and a dTAG-based PROTAC, an alternative tag technology (see Section 3), have been conducted to assess their effectiveness in degrading fused endogenous elongin BC and Polycomb repressive complex 2–associated protein (EPOP). The findings revealed that both PROTACs had a similar degradation rate; however, dTAG-based PROTACs showed a “hook effect”, where degradation efficiency reduced at higher concentrations [89].

HaloPROTAC E, comprising a chloroalkane conjugate of high-affinity VHL binder VH298, is another well-validated construct (Figure 4b) [69]. HaloPROTAC E has been shown to induce the rapid and complete degradation of HaloTag-fused serum and glucocorticoid kinase-3 (SGK3) and Class III PI3-kinase VPS34 with DC_50_ values of 3–10 nM.

**Figure 5:**
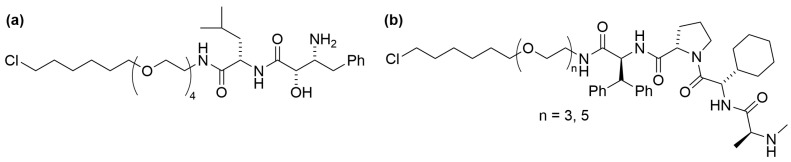
Structures of HaloPROTACs containing hexyl chloride connected to (**a**) BE04 and (**b**) MV1 E3 ligase ligands, respectively.

In addition, the HaloPROTAC concept has been expanded to recruit the E3 ligase cellular inhibitor of apoptosis protein 1 (cIAP1) by using bestatin (BE) derivatives, such as BE04, as the E3 ligase ligand (Figure 5a). This construct has been utilized to showcase the targeted ubiquitination and degradation of nuclear proteins CREB1 and c-jun fused with HaloTag [90]. Subsequently, the synthesis and biological evaluation of novel hybrid compounds consisting of a hexyl chloride moiety linked to the inhibitor of apoptosis protein (IAP) antagonist MV1 through a variety of PEG linkers have also been reported (Figure 5b) [68]. These variants were found to be more effective in reducing the levels of HaloTag-fused tumor necrosis factor α (HaloTag-TNFα) and cell division control protein 42 (HaloTag-Cdc42) compared to the BE04-linked compound.

**Figure 6:**
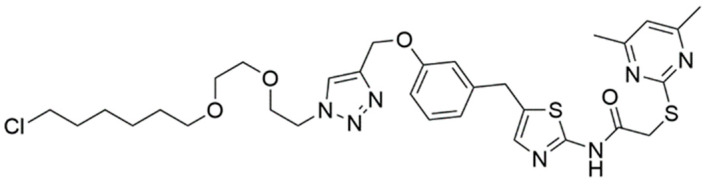
Structure of chloroalkylated SirReal.

Other adaptations of the HaloPROTAC framework have also been explored. Craig and co-workers investigated the potential for the universal applicability of E3 ubiquitin ligases in TPD [91]. They assembled a small array of HaloTag-fused E3 ligases, representing the three major classes: U-box, Homologous to the E6-AP Carboxyl Terminus (HECT), and Really Interesting New Gene (RING). Regarding the model target for degradation, they fused a F36V mutant of human FKBP (FKBP^F36V^) with EGFP (hereafter referred to as EGFP-FKBP^F36V^; for an introduction to FKBP^F36V^ tag, see Section 3.1). This system offered the benefit of a fluorescent reporter mechanism enabling high-throughput, flow-cytometry-based screening for protein degradation. In this preliminary study, the HaloTag construct of Beta-Transducin Repeat Containing Protein (βTrCP) E3 ligase demonstrated potent and efficient degradation with certain linker lengths and compositions. On the other hand, parkin E3 ligase exhibited moderate efficiency but showed broad responsiveness to all HaloPROTACs tested. Such HaloTag fusion proteins have also been utilized to develop Sirtuin-targeted PROTACs (termed SirReal, Figure 6) [92]. Quantitative analysis revealed distinct concentration-dependent effects on these HaloTag-E3 ligases of interest. Notably, HaloTag-parkin proved to be the most efficient, achieving substantial degradation of Sirt2 at a 20 nM concentration of chloroalkylated SirReal.

This approach highlights the broad applicability of the HaloPROTAC concept and sheds light on the potential roles of E3 ligases beyond CRBN, VHL, and IAPs in TPD. However, a notable concern is the possibility of losing catalytic activity due to the irreversible binding of hexyl chloride to HaloTag, which may necessitate the use of concentrations exceeding stoichiometry. Despite this challenge, HaloPROTAC’s capacity to enable the exploration of new POIs using tagging systems, without the need to develop POI specific ligands, remains a significant advantage.

### 2.3. HaloTag-Assisted Targeted Protein Dephosphorylation

Phosphorylation represents one of the most prevalent forms of PTM, occurring primarily on serine (Ser), threonine (Thr), and tyrosine (Tyr) residues. This modification entails the addition of a phosphate group, which introduces a negative charge and significantly increases the hydrophilicity of the POIs. Consequently, the altered microenvironment can precipitate changes in their structure, function, and interactions with surrounding molecules [85].

**Figure 7:**
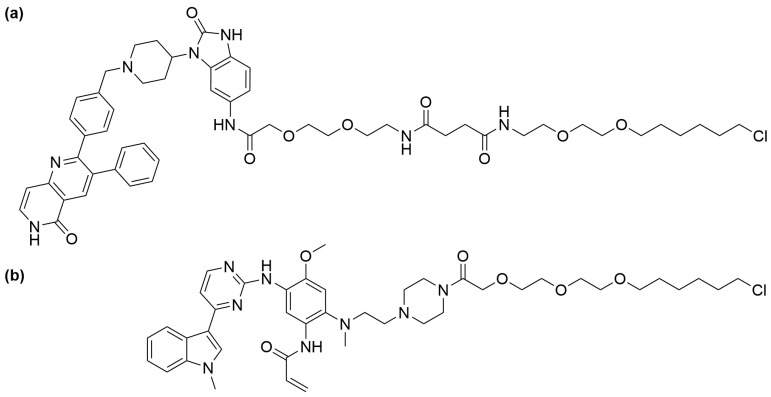
PHORCs that induce the dephosphorylation of (**a**) AKT and (**b**) EGFR using HaloTag-PP1.

HaloTag-targeted constructs of phosphatase-recruiting chimeras (PHORCs) have been developed in parallel with the peptide-based constructs [85]. The first series of PHORCs recruited HaloTag-fused protein phosphatase 1 (PP1) to induce the dephosphorylation of protein kinase B (AKT) and epidermal growth factor receptor (EGFR) (Figure 7) [85]. These constructs were extensively validated across various cell lines, demonstrating a 50–80% reduction in target-specific phosphorylation, a significant improvement over merely activating the PP1 phosphatase.

**Figure 8:**
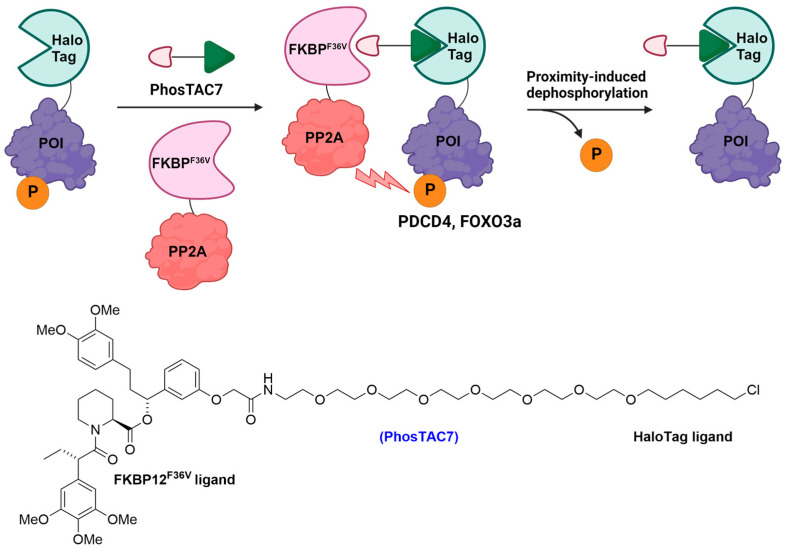
Proximity-induced dephosphorylation of HaloTag-PDCD4/HaloTag-FOXO3A by PhosTAC7.

The Crews lab also introduced a strategy, distinct from the non-selective activation of phosphatase activity, known as PhosTACs (phosphorylation-targeting chimeras), where FKBP^F36V^ was fused to protein phosphatase 2A (PP2A), while HaloTag was integrated with the POI (Figure 8) [93]. Their proof-of-concept studies involved targeting the phosphorylation events occurring on programmed cell death 4 (PDCD4) and Forkhead-box O3a transcription factor (FOXO3a) proteins [93], which utilized PhosTAC7 to remove the phosphate groups on Ser67 of HaloTag-PDCD4 and Ser318/321 of HaloTag-FOXO3A (Figure 8) [93].

### 2.4. HaloTag-Targeted Phosphorylation

**Figure 9:**
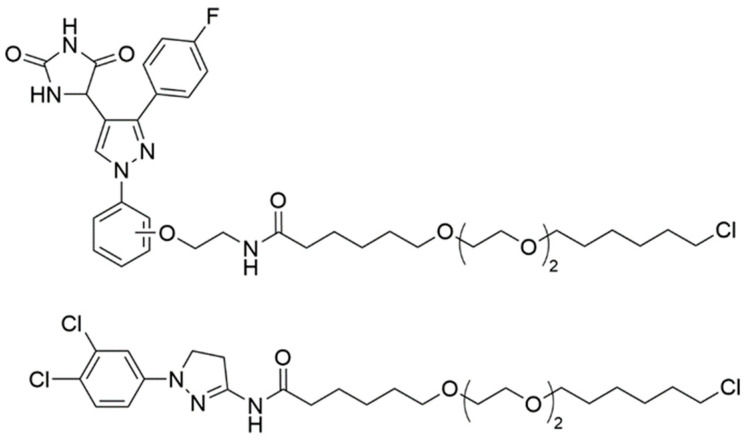
Structures of HaloTag-PHICS constructs developed by Pergu et al. [84].

Pergu et al. successfully utilized the HaloTag protein to demonstrate the concept of phosphorylation-inducing chimeric small molecules (PHICS) [84]. This method entailed the combination of hydantoin and dihydropyrazole, known binders of the Tyr kinase ABL, with chimeras featuring an extended hexyl chloride moiety (Figure 9). The authors verified the cell membrane permeability of these compounds and their efficacy in labeling free HaloTag within cells through a competition assay, where HaloTag-expressing cell lysates pre-treated with the PHICS constructs did not bind to tetramethylrhodamine (TMR)-hexyl chloride. An increase in Tyr phosphorylation on HaloTag in the presence of active PHICS, as opposed to control samples, was evidenced through Western blot analysis. However, the study did not explore the potential of the HaloTag fusion system for studying targeted phosphorylation mediated by PHICS molecules.

### 2.5. HaloTag-Assisted Transcription Factor Degradation

Crews and colleagues pioneered an innovative method to target TFs [94], which are notoriously difficult to degrade due to their elusive binding sites [95]. By taking advantage of the intrinsic TF DNA-binding ability, they devised a technique called transcription factor targeting chimeras (TRAFTAC) [94]. This method involves creating a hybrid of a double-stranded DNA, specifically recognized by the targeted TF of interest (TOI), with CRISPR-RNA. This hybrid is subsequently recognized by an ectopically expressed HaloTag-dCas9 fusion protein [94,95]. Incubation with a HaloPROTAC recruits the VHL-E3 ligase to the vicinity of the DNA-bound TOI via the HaloTag-dCas9 fusion protein, inducing the ubiquitination and proteasomal degradation of the TOI. In these proof-of-concept studies, the authors demonstrated that the TRAFTAC system can effectively induce the degradation of disease-relevant TFs, including Nuclear Factor κB (NF-κB) and Brachyury.

**Figure 10:**
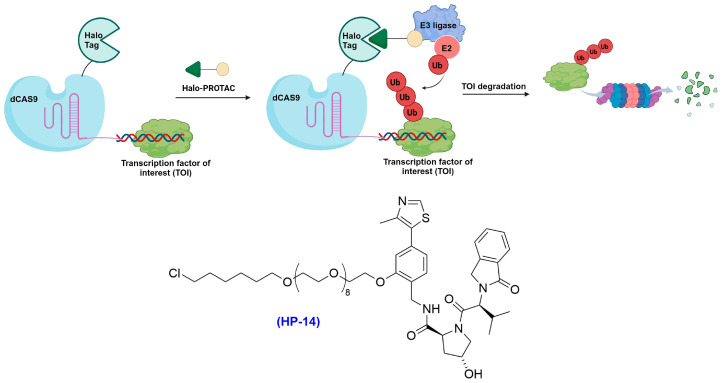
TRAFTACs mediate degradation of TFs exploiting HaloPROTACs such as HP-14.

To ensure specificity and to minimize the undesired degradation of dCas9, a comprehensive library of HaloPROTACs was screened. It began with using existing HaloPROTACs and several newly synthesized HaloPROTACs to select the optimal linker length and composition that spares dCas9 with a C-terminal HaloTag (termed CT-dCas9-HaloTag) from degradation. HaloPROTACs that have a linker with three or four PEG units were found to induce CT-dCas9-HaloTag degradation at 2.5 µM, while HaloPROTACs consist of longer linkers, such as 7-, 9- and 12-PEGylated linkers (namely HP13, HP14 and HP15 and HP16; the structure of HP14 is shown in Figure 10), did not significantly induce degradation at the same concentration [94]. Interestingly, further investigations revealed that the C-terminal HaloTag is more susceptible to degradation compared to fusing HaloTag at the N-terminus of dCas9. This finding highlights the significant impact of the HaloTag’s position within the fusion construct on both the selectivity and efficiency of targeted ubiquitination. It underscores additional factors that can be strategically manipulated to enhance the precision of selective TPD mediated by chimeric constructs.

In model studies on HP14, the authors successfully showcased the degradation of NF-κB and Brachyury in zebrafish embryos. This led to significant phenotypic alterations, most notably in the development of tail defects. These observations are consistent with the known role of Brachyury in embryonic development, specifically in aspects of migration, invasion, and metastasis [94]. Analogous to PROTACs, TRAFTACs may catalyze degradation by binding to another molecule of TOI after the initial degradation event. However, the challenge lies in effectively delivering the three components of the TRAFTAC system—HaloTag-dCas9, TRAFTAC, and HaloPROTAC—into the same cell, which may limit its application to a wider range of biological systems [95].

### 2.6. HaloTag-Assisted Recruitment of the Autophagy–Lysosome System

**Figure 11:**
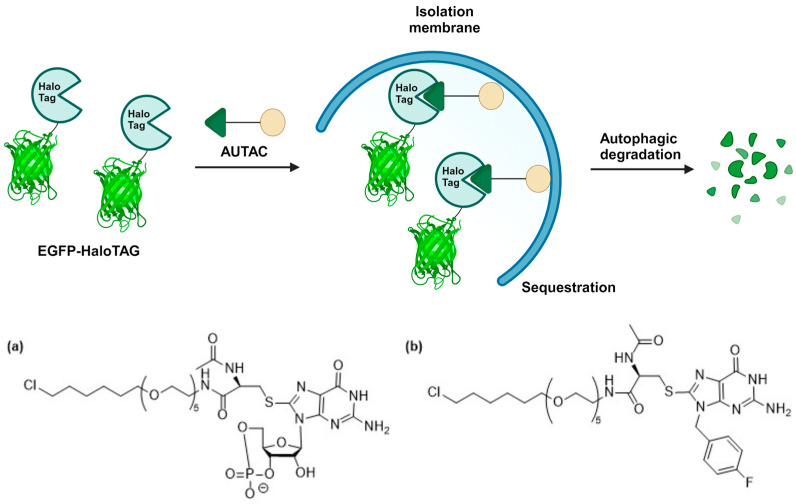
AUTAC protein degradation mechanism and structures of the autophagy tag (**a**) HaloTag-cGMP and (**b**) HaloTag-FBnG.

AUTAC represents a novel category of TPD molecules, designed to co-opt the cell’s endogenous autophagy machinery—traditionally used for the degradation of organelles and cellular debris—for the targeted degradation of POIs. The authors previously showed that S-guanylation of invading cytoplasmic group A streptococci by the endogenous nucleotide 8-nitroguanosine 3′,5′-cyclic monophosphate leads to robust K63-linked polyubiquitination and selective autophagy [62]. As such, the initial AUTAC prototypes utilized a cysteine (Cys) S-guanylation motif, connected with a hexyl chloride moiety through a PEG5 linker (Figure 11a), to guide the formation of autophagosomes and degradation of HaloTag-POIs, such as HaloTag-EGFP [62].

While the initial approach in employing cGMP-based probes for EGFP degradation showed promise, several challenges emerged. These included the slow reaction between the probe and the HaloTag fusion protein, unintended activation of cGMP-dependent protein kinase G, and less-than-ideal physicochemical characteristics. These hurdles necessitated the development of an alternative strategy, culminating in the synthesis of a novel analogue, the *p*-fluorobenzylguanine ligand (FBnG, Figure 11b). The innovative chimeric molecule, FBnG-HaloTag ligand (FBnG-HTL), displayed a markedly faster reaction with HaloTag, resulting in improved degradation kinetics and a significant reduction in off-target effects compared to its cGMP-based predecessor [62]. In practical applications with HeLa cells expressing EGFP-HaloTag, treatment with FBnG-HTL led to the formation of distinct EGFP dots. These dots notably colocalized with autophagy markers microtubule-associated protein 1 light chain 3 beta (LC3B), protein sequestosome 1(p62/SQSTM-1), and K63-linked ubiquitin chains, while the targeted degradation disappeared in autophagy-deficient Atg5^−/−^ cells and in p62 mutant mouse embryonic fibroblasts (MEF) cells. This evidence corroborates the concept of TPD via the autophagy pathway.

In exploring the potential of S-guanylation in targeted mitochondrial clearance (mitophagy), HeLa cells were engineered to stably express a fusion protein which linked the outer mitochondrial membrane (OMM) protein 25 with EGFP-HaloTag (mito-EGFP-HaloTag) and employed FBnG-HTL to label the OMM. Through immunocytochemistry, a notable colocalization of the autophagy marker LC3B with the mitochondria was observed in cells treated with FBnG-HTL. Interestingly, K63-linked polyubiquitination along the mitochondrial network was more prevalently detected than K48-linked ubiquitination. However, mitochondrial protein levels remained unchanged upon treatment with FBnG-HTL. These findings suggest that, under the given experimental conditions, S-guanylation alone may not be sufficient to significantly accelerate the process of mitophagy. However, FBnG-HTL demonstrated a notable efficacy in accelerating the clearance of fragmented mitochondria and promoting the generation of functionally intact mitochondria in fibroblast cells.

The AUTAC platform has also demonstrated successful protein degradation across a range of targets, including methionine aminopeptidase 2 (MetAP2), FKBP, BRD4, and mitochondrial translocator protein [62]. These achievements highlight the promising potential of AUTACs in precisely targeting specific proteins for degradation via autophagy. However, the mechanisms of autophagy are less understood compared to the well-established UPS. Additionally, improving the rate of degradation is critical, as current AUTACs act more slowly (often taking several hours) [62] compared to PROTACs, which usually function within an hour [96]. The efficacy of AUTACs in vivo also remains a key area for exploration.

## 3. FKBP^F36V^ Tag-Assisted Protein Modifications

### 3.1. FKBP^F36V^ Mutant

**Figure 12:**
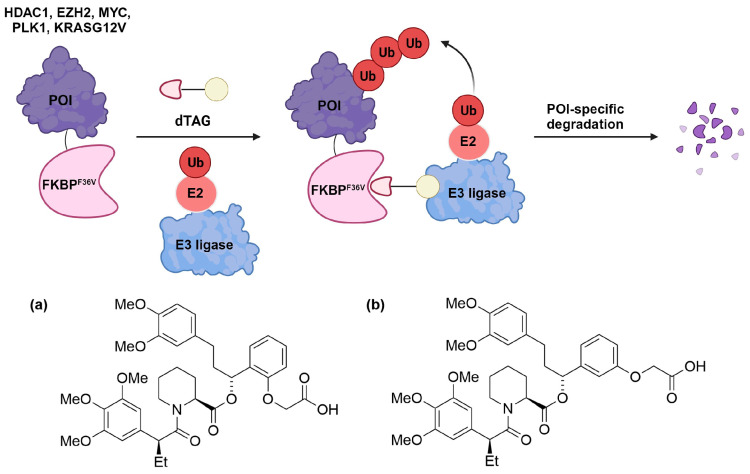
General concept of protein degradation mediated by FKBP^F36V^ and dTAG molecules consisting of (**a**) *ortho*- and (**b**) *meta*-AP1867 FKBP^F36V^ binders.

dTAG technology is centered on targeting a specific 12-kDa cytosolic prolyl isomerase F36V mutant of FKBP, that is, FKBP^F36V^, serving as the protein tag of a POI [70,97,98]. The significant feature of the mutant is its ability to create a distinct “hole” in the FKBP structure, enabling precise recognition by a synthetic ligand known as AP1867 (Figure 12) [70,99], which exhibits low affinity for the FKBP^WT^ protein. In principle, FKBP^F36V^-targeting chimera molecules are catalytic in nature and thereby enable targeted protein modification at substoichiometric concentrations. Despite this attractive feature, unlike the HaloTag fusion system, plasmids encoding FKBP^F36V^-fused human proteins are not commercially available, and the preparation of the ligand for FKBP^F36V^ demanded more labor-intensive efforts compared to that for Halo-Tag. These limitations hamper their convenience and efficiency as a model for novel targets.

### 3.2. FKBP^F36V^-Mediated Degradation

**Figure 13:**
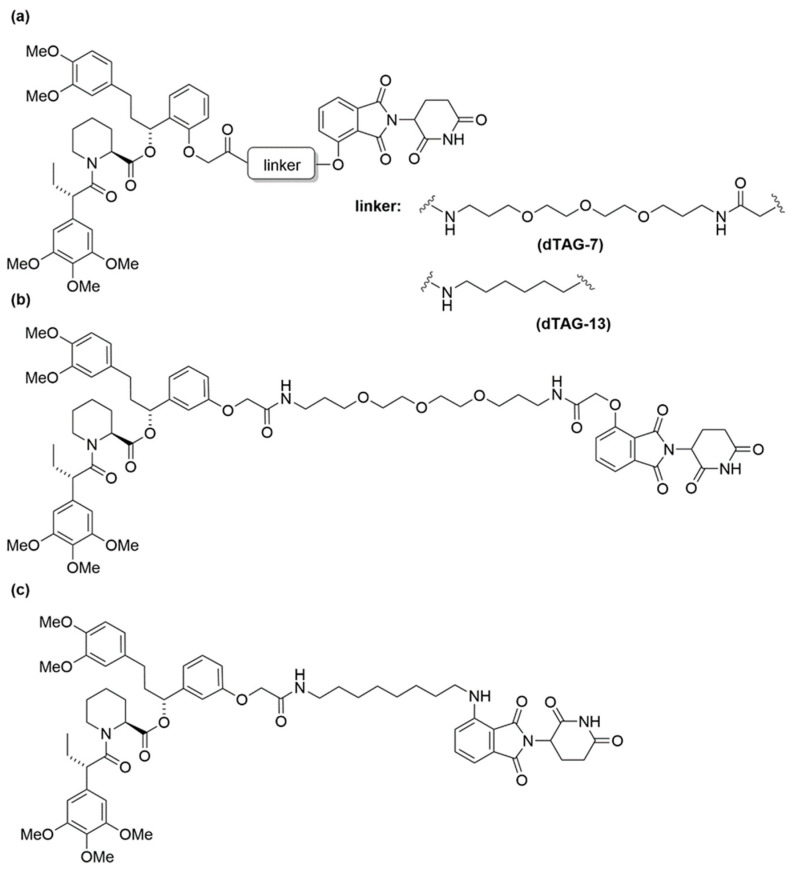
Structures of (**a**) dTAG-7, dTAG-13, (**b**) dTAG-48, and (**c**) dTAG-51.

The seminal work by Nabet et al. introduced the dTAG system, which combines (*ortho*- or *meta*-) AP1867 with a ligand that recruits the CRBN E3 ligase via various linkers [70]. These linkers strategically connect either *o*-AP1867 or *m*-AP1867, leading to the development of dTAG-7 and dTAG-13 from *o*-AP1867, and dTAG-48 and dTAG-51 from *m*-AP1867, as illustrated in Figure 13 [70]. To assess the selectivity of these heterobifunctional degraders, AlphaScreen assays were employed to evaluate their relative affinities for FKBP^WT^, FKBP^F36V^, and CRBN E3 ligase complex. The analysis revealed that dTAG-7 and dTAG-13 exhibited high specificity and submicromolar activity towards FKBP^F36V^ and CRBN. In contrast, the *m*-substituted molecules, dTAG-48 and dTAG-51, despite being highly potent in engaging FKBP^F36V^ and CRBN, demonstrated considerable affinity for FKBP^WT^ [70]. The dTAG system’s effectiveness extended to the selective degradation of various FKBP^F36V^-fused proteins, including histone deacetylase 1 (HDAC1), enhancer of zeste homolog 2 (EZH2), Myelocytomatosis (MYC), Polo-Like Kinase 1 (PLK1), and KRAS mutants, albeit with varying degradation kinetics. Notably, dTAG-13 also demonstrated rapid and potent degradation in mouse models [70].

**Figure 14:**
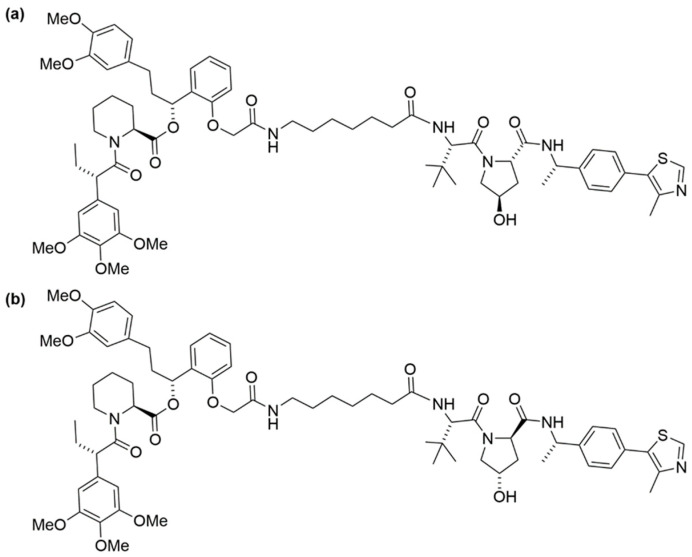
Structures of (**a**) dTAG^V^-1 and (**b**) dTAG^V^-1-NEG.

In the subsequent studies, Nabet et al. developed a new version of the dTAG system that recruits VHL E3 ligase to enhance the degradation of proteins tagged with FKBP^F36V^ [71]. Structure–activity relationship studies began with preparing *o*-AP1867-conjugated analogs with varying VHL-binding ligands and linkers. These analogs were then screened for cellular activity using 293FT FKBP^WT^-NanoLuc and FKBP^F36V^-Nanoluc dual luciferase systems. This screening culminated in the identification of dTAG^V^-1 (Figure 14a), which demonstrated potent degradation of FKBP^F36V^-Nanoluc, showing comparable efficacy to dTAG-13 and dTAG-48, while not affecting FKBP^WT^-Nanoluc.

The utility of dTAG^V^-1 was further validated through target-specific studies such as the degradation of a G12V mutant of KRAS in PATU-8902 cells expressing a FKBP^F36V^-KRAS^G12V^ fusion construct. This phenotype could be reversed by pre-treatment with the proteasome inhibitor carfilzomib or the inhibitor MLN4924 of neural precursor cell expressed developmentally down-regulated protein 8 (Nedd8) activating enzyme. Additionally, the effect was reversed in cells with a knockout of the VHL gene, which aligns with the expected mechanism of action of dTAG^V^-1. On the contrary, the diastereomer of dTAG^V^-1, namely dTAG^V^-1-NEG (Figure 14b), failed to elicit the degradation of the fused KRAS^G12V^. This outcome stemmed from the inability of dTAG^V^-1-NEG to bind and recruit VHL.

Moreover, the authors demonstrated that dTAG^V^-1 possessed excellent pharmacokinetic and pharmacodynamic properties, making it an effective tool for in vivo investigation [71]. This is supported by a recent report which successfully demonstrated the effective degradation of a core mediator subunit, mediator complex subunit 14, using dTAG^V^-1 in HCT116 cells [100]. Notably, in this context, CRBN-recruiting dTAG molecules proved ineffective. These findings collectively underscore the potential of dTAG^V^-1 to surpass some limitations of CRBN-based dTAG systems.

**Figure 15:**
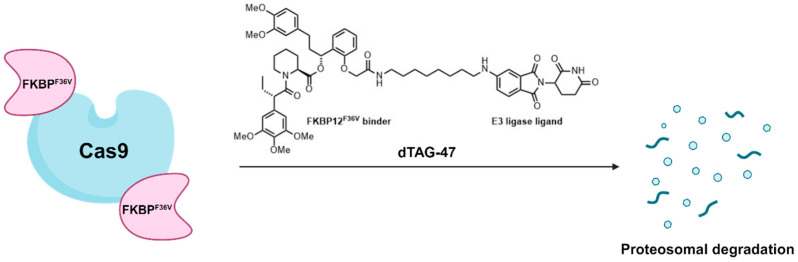
FKBP^F36V^-tagged Cas9 degradation mediated by dTAG-47.

CRISPR-associated protein 9 (Cas9), a groundbreaking RNA-guided DNA endonuclease, has been a pivotal tool in revolutionizing genome editing. However, lingering Cas9 activity can lead to unwanted consequences, such as genotoxicity and immunogenicity [99]. To address this challenge, Choudary and co-workers developed a chemo-genetic strategy leveraging the dTAG system to regulate the lifespan of Cas9 in cells [99]. By fusing the FKBP^F36V^ tag to various sites on Cas9, they strategically positioned it near a ubiquitin ligase. This setup facilitated the rapid ubiquitination and subsequent proteasome-mediated degradation of Cas9 upon ligand binding using heterobifunctional molecules that target both FKBP^F36V^ and CRBN. Furthermore, dTAG-47 has proven effective in inducing the degradation of FKBP^F36V^-fused Cas9 across species, including Drosophila and mice [99]. This study also emphasized the impact of Cas9′s longevity on DNA repair processes after double-strand breaks, underscoring the importance of precise control for genome editing outcomes and specificity (Figure 15).

### 3.3. FKBP^F36V^-Mediated Targeted Tyrosine Phosphorylation

**Figure 16:**
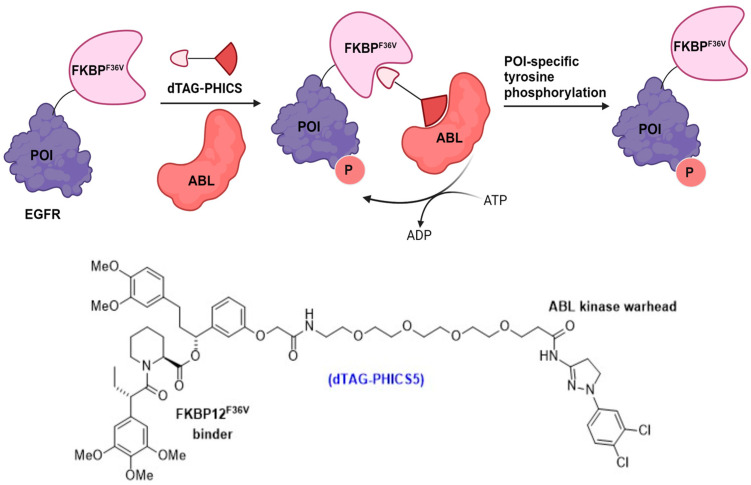
Tag-based phosphorylation inducing chimera PHICS-5 utilizing the FKBP^F36V^ tag.

The latest PHICS-based constructs have been integrated with dTAG technology, illustrating their effectiveness in studies focused on target-specific Tyr phosphorylation (Figure 16) [84]. The study began by creating a catalytically inactive variant of the EGFR’s intracellular domain, which was then fused with FKBP^F36V^, resulting in a construct named iEGFR-FKBP^F36V^-FLAG. Further analysis through immunoblotting of cell lysates, employing site-specific phosphotyrosine antibodies, indicated that ABL PHICS-5 was capable of phosphorylating Tyr residues within various sequence contexts. Importantly, PHICS-induced Tyr phosphorylation on functional EGFR was found to trigger downstream receptor signaling [84]. This process was quantitatively measured using a luciferase-based reporter gene assay sensitive to the serum response element. These findings highlight the ability of cytoplasmic ABL to target integral membrane proteins and induce functionally relevant Tyr phosphorylations across a broad spectrum of amino acid sequences, effectively leveraged through PHICS technology.

### 3.4. FKBP^F36V^-Mediated Targeted Protein Acetylation

**Figure 17:**
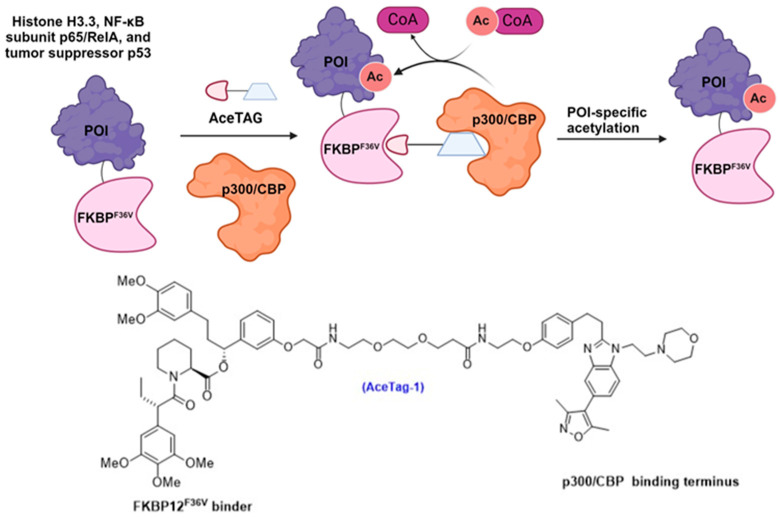
Targeted acetylation of a FKBP^F36V^-tagged POI induced by AceTAG-1.

The newly introduced acetylation-inducing chimera, termed AceTAG, also utilizes the FKBP^F36V^ tag for proof-of-concept studies (Figure 17) [87]. This innovative AceTAG system harnesses heterobifunctional molecules designed to bring the Lys acetyltransferase p300/CBP into close proximity with the POI. This proximity-induced reaction is rapid and selective, and has been shown to induce the acetylation of histone H3.3, NF-κB subunit p65/RelA, and tumor suppressor p53 [87].

## 4. BromoTag

Ciulli and colleagues pioneered the BromoTag inducible degron system, a novel approach grounded in the “bump-hole” pair concept [101]. This system incorporates a strategic amino acid mutation (L387A) within the second bromodomain of BRD4 (termed BromoTag), effectively creating a cavity (or “hole”) tailored for a bulkier synthetic bromodomain ligand bearing a “bump”. Such a unique configuration allows for the selective targeting of a “bumped” PROTAC, ensuring that the activity and abundance of the endogenous wild-type bromodomains remain unaffected.

The authors reasoned that because BRD2 bromodomains are vulnerable to degradation by standard bromodomain-targeted PROTACs, using a heterozygous knock-in cell line would enable simultaneous tracking of both on-target (BromoTagged-BRD2) and off-target (untagged BRD2) effects during degradation studies.

**Figure 18:**
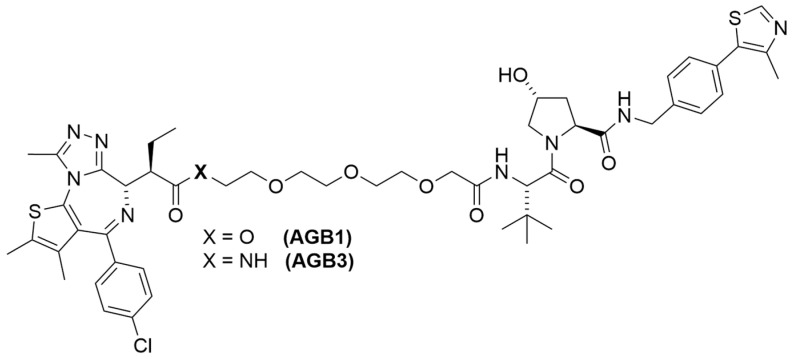
Structures of AGB1 and AGB3.

In the initial screening of BromoTag-targeted PROTACs, AGB1 emerged as a standout candidate (Figure 18). It demonstrated remarkable efficacy in forming a robust cooperative ternary complex with VHL and the BromoTag, effectively leading to the complete degradation of BromoTagged targets with low nanomolar potency. It achieves this with exceptional selectivity, sparing native wild-type bromodomain proteins across the proteome. Interestingly, when comparing the degradation efficiency of AGB1 analogs in a cellular context, the ester variant of AGB1 proved significantly more potent in on-target degradation than the amide variant of AGB1 (AGB3) [101]. A recent study exploring the effects of amide-to-ester substitutions in bromodomain-targeted PROTACs also highlighted an increase in potency, plausibly due to enhanced cellular permeability [102]. Beyond its biochemical efficiency, AGB1 also exhibits advantageous pharmacological properties. It has shown minimal cytotoxicity, robust plasma stability, and a favorable pharmacokinetic profile, making it a standout in the range of BromoTag-targeted PROTACs [101]. These properties position it favorably for future in vivo studies.

**Figure 19:**
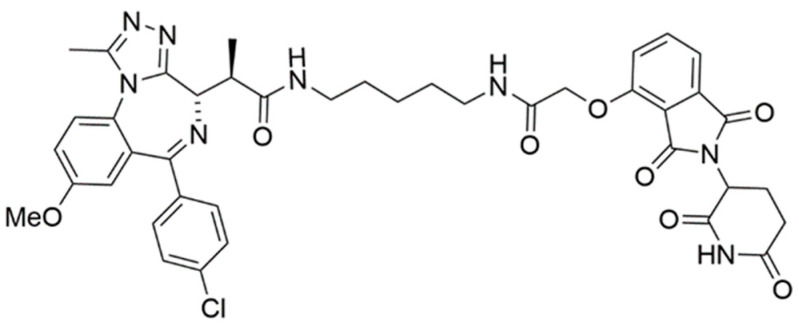
Structure of XY-06-007.

In the same year, Fischer et al. also reported a “bump-and-hole” bromodomain-based degradation tag, wherein BRD4^L94V^ was employed as the protein tag [103]. Their study also demonstrated the transformation of a BRD4 inhibitor into specific “bumped” degraders of the BRD4 mutant through recruiting CRBN E3 ligase. For example, XY-06-007 (Figure 19) exhibited exceptional efficacy with a DC_50_ of 10 nM while sparing off-target proteins from degradation, as assessed by proteomic mass spectrometry [103].

The authors also explored a dual degradation approach with the aim of providing a binary regulatory system within the same cellular context. This method was first demonstrated in cells expressing a BRD4^L94V^ degron fused with EGFP and a HiBit-FKBP^F36V^-KRAS^G12C^ fusion construct. Their study revealed that the degradation of both BRD4^L94V^-EGFP, mediated by compound XY-06-007, and HiBit-FKBP^F36V^-KRAS^G12C^, mediated by dTAG-13 (Figure 13a, Section 3.2), was minimally affected when combined with 1 µM of the respective other compound. In the subsequent studies, the authors also demonstrated that the degradation of HiBit-FKBP^F36V^-KRAS^G12C^ by dTAG^V^-1 (Figure 14a) was not influenced by the presence of 1 μM of XY-06-007; however, when the roles were reversed, the degradation of BRD4^L94V^-EGFP by XY-06-007 was significantly enhanced with the addition of 0.1–1 µM dTAG^V^-1. Interestingly, the same results can be achieved using the negative control, dTAG^V^-1-NEG (Figure 14b), suggesting that the effects observed were not driven by VHL binding or its associated ubiquitin ligase activity. This led to the hypothesis that dTAG^V^-1 might function through saturating P-glycoprotein (P-gp). The hypothesis gains support from observations where the degradation efficacy of BRD4^L94V^-EGFP was notably enhanced upon co-incubation with a P-gp inhibitor (tariquidar) at concentrations of 125 nM and 1 μM. Nonetheless, the versatility of the BromoTag concept allows it to complement other tagging systems such as FKBP^F36V^ and HaloTag. This integration fosters the potential for conditional TPD by strategically pairing specific protein-tagging systems with the corresponding chemical degraders.

## 5. NanoTAC

**Figure 20:**
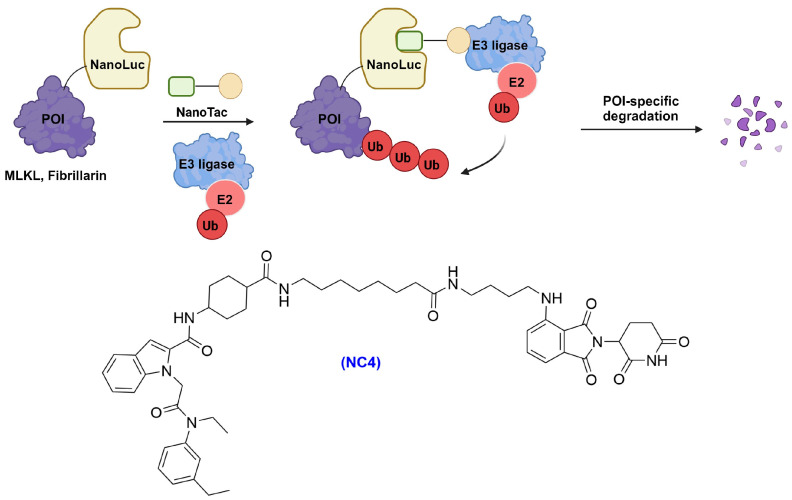
NanoLuc targeting degrader system involving NC4.

NanoLuc, a commercially available 19 kDa luciferase enzyme, emits high-intensity bioluminescence signals upon activation by furimazine [104]. Its stability, compact size, and compatibility with in vivo substrates have garnered new interest in developing it as a tag for TPD [105]. Grohmann et al. introduced NanoLuc-targeting PROTACs (NanoTACs) designed to initiate the proteasomal degradation of NanoLuc-tagged substrates, facilitating the rapid bioluminescence screening of POI degradation [106]. Two series of NanoTAC candidates targeting E3 ligase CRBN and VHL, respectively, were synthesized using varied alkyl chain linker lengths and NanoLuc-binding ligands. A detailed comparison across various protein-tagging systems and E3 ligases highlighted the degradation efficiency mediated by NanoTAC4 (NC4, Figure 20) in the NanoLuc-CRBN pairing system, comparable to the performance of HaloPROTAC and dTAG molecules in their respective systems [106]. However, the efficacy of NC4 diminished after 24 h, a factor requiring consideration in its application. The study also explored the impact of different tagging systems on test substrates such as pseudokinase Mixed-Lineage Kinase Domain-Like (MLKL) protein and Fibrillarin. It was reported that NanoLuc fusion proteins underwent effective degradation at low nanomolar concentrations with NanoTACs. However, it was noted that FKBP^F36V^ generally demonstrated superior efficiency in inducing the degradation of FKBP^F36V^-tagged substrates through recruiting CRBN E3 ligase compared to those fused with HaloTag and NanoLuc [106]. These FKBP^F36V^-based systems required not only lower effective concentrations but also less time to achieve significant protein degradation. The observed differences in efficacy between NanoTACs and dTAGs might be due to their differential distribution within various cellular compartments [106]. Although NanoTACs exhibit catalytic properties similar to dTAGs, further investigations are necessary to fully understand their compatibility in vivo.

Nevertheless, for insights into tagged protein biology, such as cellular localization or protein–protein interactions, NanoLuc and HaloTag might offer an additional advantage due to their commercial availability [106]. Overall, these studies demonstrated that efficient tag-targeted protein degradation is system-dependent, and it is essential to compare each tag against individual substrates to identify the optimal degrader/tag pair.

## 6. CH6-Tag

**Figure 21:**
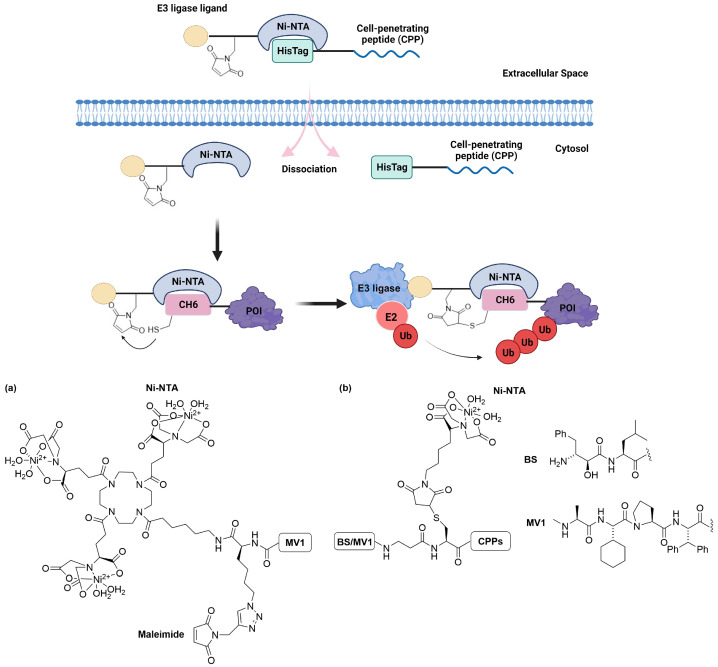
Delivery of SNIPER-CH6 to ubiquitylate and degrade CH6-tagged POI. Structures of (**a**) SNIPER(CH6) and (**b**) BS/MV1-CPP-Ni-NTa.

Okitsu et al. developed an innovative protein knockdown method leveraging nickel-chelating chemistry [107]. This approach involves fusing a POI with a CH6-tag, which typically consists of six His residues and one Cys, attached at either the N- or C-terminus of the POI. This design is carefully crafted to minimize disruption to the POI’s function [108]. The degrader system for this CH6-tagged POI incorporates two external elements: a degradation inducer named SNIPER(CH6) and a carrier peptide. SNIPER(CH6) comprises nickel nitrilotriacetic acid (Ni-NTA) for binding to the polyhistidine tag (His-tag) as part of the CH6-tag, a maleimide electrophilic functional group, and a ligand (MV1) for recruiting IAP E3 ligases (Figure 21a). To enhance cellular uptake, a cell-penetrating peptide (CPP) featuring a His-tag is also included to form a complex with SNIPER(CH6).

The degradation of CH6-tagged POIs is facilitated through a multi-step mechanism (Figure 21). Initially, the binary complex penetrates the cell, leading to the subsequent intracellular release of SNIPER(CH6). This molecule then specifically binds to the CH6-tagged POI. A critical phase in this process is the covalent interaction between the maleimide group in SNIPER(CH6) and the thiol side chain of the Cys residue within the CH6-tag. The final step involves SNIPER acting as a bridge, bringing the IAP E3 ligase into close proximity with the POI, thereby initiating targeted ubiquitination and degradation. This system has demonstrated the efficient degradation of CH6-tagged cellular retinoic acid-binding protein 2 (CRABP2) and Mothers Against Decapentaplegic Homolog 2 (Smad2) protein. However, in the absence of either the maleimide or the carrier peptide, no degradation of the POIs was observed [108].

Although the CH6-tag serves as a valuable tool, this approach faces two primary limitations [109]. First, it requires the combined action of two molecules: SNIPER(CH6) and the carrier peptide. Second, the UPS also facilitates the degradation of SNIPER(CH6) due to its covalent conjugation to the CH6-tag. This necessitates the use of suprastoichiometric quantities of SNIPER(CH6) and carrier peptide molecules to achieve complete degradation. As such, the authors devised a new series of Ni-NTA-based inducers for degrading His-tagged POIs [109]. This novel inducer is composed of Ni-NTA, an IAP antagonist such as BE or MV1, and a CPP peptide such as Tat or nonarginine (R9). This results in the formation of molecular chimeras: BE-Tat-Ni-NTA, MV1-Tat-Ni-NTA, BE-R9-Ni-NTA, and MV1-R9-Ni-NTA (Figure 21b). The evaluation of degradation was conducted on CRABP2-T7 protein with a His-tag fused at the N-terminus of CRABP2. In general, MV1-based molecules exhibited a higher degradation efficiency when compared to their BE-conjugated counterparts, especially at lower concentrations (3–5 µM) [109]. This observation is in line with prior research demonstrating the superior performance of MV1 over BE in terms of recruiting the E3 ligase [110]. Additionally, the carrier peptide R9 demonstrated greater effectiveness in transporting cargo molecules into HT1080 cells. Among all the variants, MV1-R9-Ni-NTA emerged as the most efficient inducer for the degradation of His-tagged proteins [109].

## 7. AchillesTag

Expanding upon the concept of protein tag-based degraders, a recent advancement emerged with the development of the AchillesTag (aTAG) system [111]. This system consists of two key components: a compact protein degron tag and a bifunctional molecule that activates degradation, termed BIDAC^TM^, that binds to the degron tag and the CRBN E3 ligase simultaneously to facilitate targeted ubiquitination and degradation. In their research, the investigators selected a compact protein, MuT Homolog 1 (MTH1)/Nudix Hydrolase 1 (NUDT1), as the degron tag for development. This choice was based on MTH1′s small size (approximately 18 kDa) and its structure, which features nine surface-exposed Lys residues prone to ubiquitination modifications [111].

**Figure 22:**
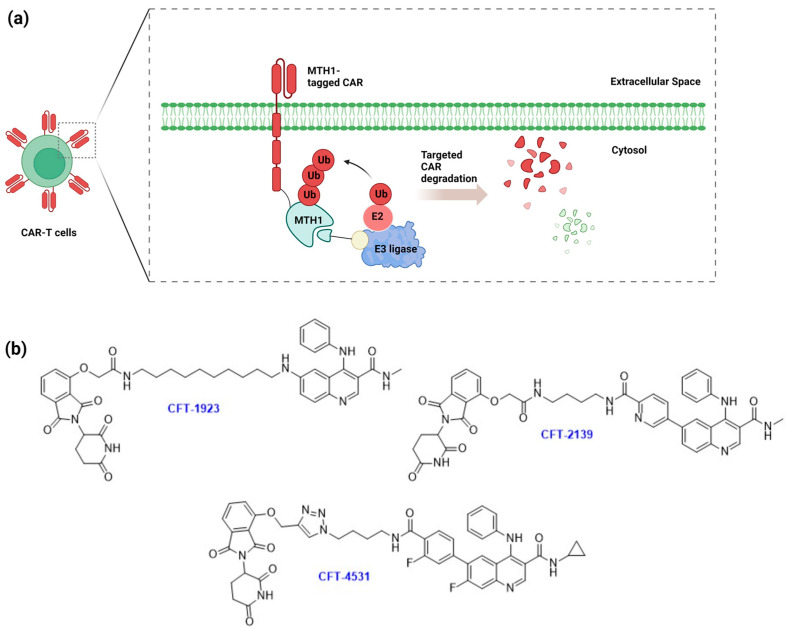
The application of aTAG system in modulating CAR expression level. (**a**) MTH1 tag is prone to be degraded via BIDAC^TM^-mediated targeted ubiquitination and degradation leading to CAR elimination on demand; (**b**) representative structures of BIDAC^TM^ used in the study.

Prior to initiating a structure–activity relationship campaign for identifying MTH1-targeting degraders, the researchers first established cell lines that express the MTH1 aTAG in fusion with a target protein. They engineered a fusion where full-length MTH1 was attached to the C-terminus of a Chimeric Antigen Receptor (CAR) aimed at the B cell antigen CD19, incorporating an epitope tag, such as HA or HiBiT, for detection and quantification. These engineered constructs were then overexpressed in human Jurkat T-cells aided by lentivirus-mediated infection. This fusion protein was named Small-Molecule-Activated Rheostat of T-cells (SMART-CAR). In the initial screening for MTH1 ligands, aminoquinazoline and quinoline scaffolds were chosen, resulting in the selection of aTAG degraders CFT-1923, CFT-2139, and CFT-4531 for comprehensive activity profiling (Figure 22). This selection was based on their demonstrated effectiveness and selectivity in recruiting CRBN E3 ligase. In the subsequent studies, SMART-CAR-expressing Jurkat T-cells were stimulated with CD19+ Raji target cells at an Effector/Target ratio of 1:3, leading to potent target cell elimination. The authors also demonstrated a concentration-dependent degradation of SMART-CAR in this process.

Following a single dose of CFT-1923, CFT-2139, or CFT-4531, which achieved complete SMART-CAR degradation, cell washout experiments showed that the SMART-CAR protein recovered to the basal level over 72 h. Therefore, the researchers suggested that this method can provide a functional CAR-T rheostat switch. In subsequent studies, no degradation was observed with native mouse MTH1 and a lack of dose–response degradation was also observed in the B16F10 mouse melanoma cell line, indicating that mouse MTH1 may be inaccessible for aTAG applications. Nonetheless, the study verified the effectiveness of these degraders in degrading human proteins, highlighting the potential of such technology in studying human diseases.

## 8. SpyTag-SpyCatcher-Mediated Targeted Degradation

**Figure 23:**
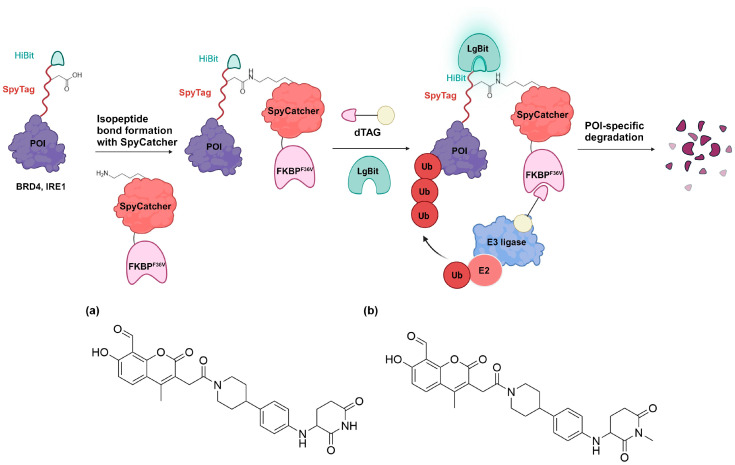
SpyCatcher-dTAG binds covalently to HiBiT-SpyTAG fused protein, allowing recruitment of E3 ligase to degrade the fused POI. Structures of IRE1α PROTACs (**a**) CPD-2828 and (**b**) CPD-3121.

Tsang et al. developed a series of concise 24−25 amino acid tags collectively termed HiBiT-SpyTag [112]. HiBiT-SpyTag is constructed by combining the HiBiT peptide (VSGWRLFKKIS) for precise protein quantification with SpyTag [113], a tag known for its ability to spontaneously form an isopeptide bond with SpyCatcher protein. HiBiT-SpyTag was designed to enable covalent protein capture through interaction with FKBP^F36V^-SpyCatcher co-expressed in the same system [113]. This innovative system enables the use of existing dTAG molecules to facilitate HiBiT-SpyTag-labeled POIs (Figure 23). The HiBiT-SpyTag peptide can be strategically placed at the N-terminus, or alternatively, SpyTag-HiBiT can be positioned at the C-terminus of the POI. Both fusion strategies facilitate the application of HiBiT-based luminescence assays for the accurate quantification of protein concentrations.

A notable application involved endogenously tagging the BRD4 locus at the N-terminus with HiBiT-SpyTag002 through CRISPR-Cas9 engineering [112]. Transfecting FKBP^F36V^-SpyCatcher subsequently induced efficient protein degradation through dTAG13. In contrast, the control PROTAC dBET6, a BRD4-targeting PROTAC, degraded BRD4 equally with or without the presence of FKBP^F36V^-SpyCatcher. Expanding on this proof-of-concept, the authors applied their methodology to inositol-requiring enzyme 1 α (IRE1α), a previously unreported degradable protein, mediating the unfolded protein response. A SpyTag-HiBiT knock-in HEK293T cell line was generated by fusing the C-terminus of IRE1α with the peptide tag. A dose–response degradation assay with dTAG13 achieved a potent degradation of IRE1α-SpyTag-HiBiT, providing a D_max_ of 61–70% [112]. Furthermore, comparative studies with a CRBN-based PROTAC (CPD-2828) of inositol-requiring transmembrane kinase/endoribonuclease 1α (IRE1α) were also conducted. CRBN PROTAC CPD-2828 (Figure 23a) was found to selectively degrade endogenous IRE1α in cells, while the negative control CPD-3121 did not (Figure 23b).

In summary, integrating HiBiT and SpyTag technologies provides precise control and detection of protein expression. In theory, this system’s modularity could be further expanded by incorporating protein localization tags into FKBP^F36V^-SpyCatcher [114]. Such an addition would facilitate the selective depletion of a particular pool of the target protein. This potential for targeted manipulation within distinct cellular contexts underscores the versatility and applicability of this integrated approach.

## 9. Conclusions

In conclusion, the burgeoning field of proximity-inducing chimeras, exemplified by PROTAC technology, represents a transformative shift in studying protein modifications within living systems. These agents harness protein modification machineries to precisely manipulate the functional states of POIs, opening new avenues for targeting disease-related proteins. Despite their significant promise in pre-clinical and clinical settings, these technologies encounter significant hurdles, such as limited ligand availability for the extensive human proteome, suboptimal ligand selectivity raising the risk of off-target effects, limited control over the timing, tissue specificity, and spatial distribution of the bifunctional molecules.

Protein tag-mediated targeted protein modification presents a viable alternative to overcome these challenges both in vitro and in vivo. This approach provides a complementary option to common genetic knockdown/knockout methods such as CRISPR/Cas9 and RNA interference. It enables highly selective engagement with the POI without needing to develop specific ligands from scratch. Moreover, the genetic engineering basis of these tagging systems enables temporal control over protein levels and localization, with some systems now commercially available and linked to the human proteome. However, selecting the protein tag for a specific POI poses a challenge due to the lack of a definitive guide for choosing the optimal tag, with the risk that a large tag might disrupt protein function or protein–protein interactions. Continued development in degron tag knock-ins for in vitro and in vivo validation is essential to address this concern. In addition, the bifunctional molecules designed for this approach may not be suitable as therapeutic agents, although they remain valuable tools for researching animal physiology and pathology using knock-in models. The other features and their associated advantages and disadvantages have been summarized in Table 1.

Among the current array of protein tag technologies, the FKBP^F36V^ tag, recruited by the synthetic ligand AP1867, emerges as a promising pair for protein modification studies, demonstrating minimal off-target effects. Its singular genetic insertion requirement and proven in vivo efficacy position it as a highly promising candidate. Similarly, HaloTag also presents a compelling solution for targeting POIs, utilizing a hexyl chloride-containing chimera molecule. Both FKBP^F36V^ tag and HaloTag have showcased their potential in enabling functional studies of human proteins lacking existing binders. This capability has become integral in developing chemical-induced PTM approaches, such as PHICS, PHORCS, and AceTAC. Looking forward, efforts in developing protein tags with smaller molecular weights become imperative to minimize perturbations in protein folding and function. As these methodologies continue to evolve, they hold the promise of revolutionizing our understanding of the hidden functions of PTMs and addressing a wide range of biological and medical questions.

## Figures and Tables

**Table 1:** Summary of different tag degrader technology.

Tag	Tag Size (kDa)	Examples of Degraders	Catalytic Degraders	Orthogonality of Tag	Luminescence Properties	In Vivo Application	Advantages/Disadvantages
HaloTag	33	HaloPROTAC3, HaloPROTAC E, SirReal, PHORCs, HaloPHICS, HaloPROTAC14	✗	✓	✗	✓	It requires only a single genetic construct. HaloTag features a rapid, specific, and irreversible reaction with a hexyl chloride-containing chimera. Plasmids encoding HaloTag-fusion human proteome are commercially available. HaloPROTACs are non-catalytic and require suprastoichiometric amounts for full occupancy. The large size of the HaloTag may affect the folding, function, and activity of POIs.
FKBP^F36V^	12	dTAG-V1, dTAG-13, PhosTAC7, dTAG-PHICS, AceTAG-1	✓	✓	✗	✓	Compact size. FKBP^F36V^-targeting chimeric molecules function at substoichiometric concentrations. High selectivity and rapid degradation of POIs. Excellent pharmacokinetic/pharmacodynamic properties. Plasmids encoding FKBP^F36V^-fusion human proteome are not commercially available. Degradation rates depend on subcellular compartments [114].
BromoTag	15	AGB1, XY-06-007	✓	✓	✗	✓	Compact size. Highly selective, no off-target degradation of endogenous BET proteins. Minimal cytotoxicity. Rapid kinetics. Suitable for in vivo studies due to robust plasma stability and a favorable pharmacokinetic profile.
NanoLuc	19	NC4	✓	✓	✓	✗	Compact size. Compatibility with in vivo substrates. The efficiency of NanoLuc-mediated targeted degradation varies with the choice of E3 ligase and ligands. Only VHL and CRBN E3 ligases have been recruited for degrading NanoLuc-tagged POIs.
CH6-tag	0.8	SNIPER-CH6, BS/MV1-CPP-Ni-NTA	✗	✓	✗	✗	Smallest in size among all the protein tags used in proximity-induced TPD studies. The absence of Lys residues in this tag can avoid complications that arise in larger tags, which may lead to premature degradation due to ubiquitination. Small-molecule-based degraders targeting CH6-tag need an additional CPP component. Potential non-specific binding issues with Ni-NTA in cells. The toxicity of Ni-NTA-based constructs requires further investigation.
aTAG	19	aTAG-1923, aTAG-2139, aTAG-4531	✓	✓	✗	✓	Compact size with potent and rapid binding capabilities. Mouse MTH1 does not respond to current aTAG constructs. Only CRBN E3 ligase has been recruited for SMART-CAR constructs.
HiBiT-SpyTag	24–25 amino acids	dTAG-13	✓	✓	✓	✗	Short peptide tags. Allows for the quantification and covalent functionalization of fused proteins at late stage. It requires co-expression of SpyCatcher-containing protein tag in order to facilitate targeted protein modulation. Due to its size, the covalent adduct formed with SpyCatcher may impact the function of POI.

## Abbreviations

**AceTAG**, acetylation-inducing chimera; **AID**, auxin-inducible degron; **AKT**, protein kinase B; **Asp**, aspartate; **aTAG**, AchillesTag; **ATTECs**, autophagosome-tethering compounds; **AUTACs**, autophagy-targeting chimeras; **BE**, bestatin; **βTrCP**, beta-transducin repeat containing protein; **CAR**, chimeric antigen receptor; **Cas9**, CRISPR-associated protein 9; **Cdc42**, cell division control protein 42; **cIAP1**, cellular inhibitor of apoptosis protein 1; **CIP**, chemically induced proximity; **CPP**, cell-penetrating peptide; **CRABP2**, cellular retinoic acid-binding protein 2; **CRBN**, protein cereblon; **CT-dCas9-HaloTag**, dCas9 with a C-terminal HaloTag; **Cys**, cysteine; **DC_50_** half-maximal degradation concentration; **∆F508-CFTR**, deletion of Phe508 of cystic fibrosis transmembrane conductance regulator; **dTAG**, degradation tag; **DUBTAC**, deubiquitinase-targeting chimera; **EGFP**, enhanced green fluorescent protein; **EGFR**, epidermal growth factor receptor; **EPOP**, endogenous elongin BC and Polycomb repressive complex 2–associated protein; **ERK1**, extracellular signal-regulated protein kinase 1; **EZH2**, enhancer of zeste homolog 2; **FBnG**, *p*-fluorobenzylguanine ligand; **FKBP**, FK506-binding protein; **FKBP^F36V^**, F36V mutant of human FKBP; **FOXO3a**, Forkhead-box O3a transcription factor; **Gly**, glycine; **HaloPROTACs**, HaloTag-targeted PROTACs; **HaloTag-TNFα**, HaloTag-fused tumor necrosis factor α; **HDAC1**, histone deacetylase 1; **HECT**, homologous to the E6-AP carboxyl terminus; **His**, histidine; **IAPs**, inhibitor of apoptosis proteins; **IRE1α**, Inositol-requiring transmembrane kinase/endoribonuclease 1α; **KineTACs**, cytokine-receptor-targeting chimeras; **LC3B**, microtubule-associated protein 1 light chain 3 beta; **Lys**, lysine; **LYTACs**, lysosome-targeting chimeras; **MEF**, mouse embryonic fibroblasts; **MEK1**, MAP/ERK kinase 1; **MetAP2**, methionine aminopeptidase 2; **MLKL**, mixed-lineage kinase domain-like; **MTH1**, MuT homolog 1; **MYC**, myelocytomatosis; **NanoTACs**, NanoLuc-targeting PROTACs; **Nedd8**, neural precursor cell-expressed developmentally down-regulated protein 8; **Ni-NTA**, nickel nitrilotriacetic acid; **NUDT1**, nudix hydrolase 1; **OMM**, outer mitochondrial membrane; **OTUB1**, OTU deubiquitinase, ubiquitin aldehyde binding 1; **p62/SQSTM-1**, protein sequestosome 1; **PDCD4**, phosphorylation events occurring on programmed cell death 4; **P-gp**, P-glycoprotein; **Phe**, phenylalanine; **PHICS**, phosphorylation-inducing chimeric small molecules; **PHORC**, phosphatase-recruiting chimera; **PhosTAC**, phosphorylation-targeting chimera; **PLK1**, polo-like kinase 1; **POI**, protein of interest; **PP1**, protein phosphatase 1; **PP2A**, protein phosphatase 2A; **PPIs**, protein–protein interactions; **PROTAB**, proteolysis-targeting antibody; **PROTAC**, proteolysis-targeting chimera; **PTMs**, post-translational modifications; **R9**, nonarginine; **RING**, really interesting new gene; **Ser**, serine; **SirReal**, sirtuin-targeted PROTACs; **Smad2**, mothers against decapentaplegic homolog 2 protein; **SMART-CAR** small-molecule-activated rheostat of T-cells; **TF**, transcription factor; **Thr**, threonine; **TIR1,** transport inhibitor response 1; **TMR**, tetramethylrhodamine; **TOI**, transcription factor of interest; **TPD**, targeted protein degradation; **TRAFTAC**, transcription factor-targeting chimeras; **Tyr**, tyrosine; **UPS**, ubiquitin–proteasome system; **VHL**, von Hippel–Lindau disease tumor suppressor.
